# *ESR1* dysfunction triggers neuroinflammation as a critical upstream causative factor of the Alzheimer’s disease process

**DOI:** 10.18632/aging.204359

**Published:** 2022-11-01

**Authors:** Junying Liu, Shouli Yuan, Xinhui Niu, Robbie Kelleher, Helen Sheridan

**Affiliations:** 1 NatPro Center, School of Pharmacy and Pharmaceutical Sciences, Trinity College Dublin, Dublin 2, Ireland; 2 Academy for Advanced Interdisciplinary Studies, Peking University, Beijing, China; 3 Department of Regenerative Medicine, School of Pharmaceutical Sciences, Jilin University, Changchun, China; 4 School of Medicine, Trinity College Dublin, Dublin 2, Ireland

**Keywords:** *ESR1*, Alzheimer’s disease, *CEBPB/ATF4*, *APOE*, pyroptosis

## Abstract

Alzheimer's disease (AD) accounts for approximately 60% of dementia cases worldwide. Advanced age is the most significant risk factor for AD and approximately two-thirds of cases relate to women. While the previous meta-analysis suggests that estrogen receptor (ESR) genetic polymorphisms are closely associated with dementia, the implications of this observation on a molecular level are not entirely understood. Our study explores this intricate molecular puzzle through the use of a variety of bioinformatics tools. Initially, we attempted to elucidate mechanisms underlying breast cancer development by identifying the high-throughput dataset of *ESR1*-knockdown breast cancer tissue samples. Surprisingly, KEGG pathway enrichment showed that the most frequently occurring proteins were related to axonal guidance and inflammation-related gene markers. These observations were supported by an external high throughput dataset of AD inflammatory samples *in vivo*. Our results suggest that *ESR1* is modulated by apolipoprotein E (*APOE*) through *CEBPB/ATF4*, mir-155-5p, or mir-1-3p. Moreover, sea hare-hydrolysates (SHH), as one of the axonal guidance molecules, could regulate the STAT3/PRDM1/CEBPB pathway and consequently induce cell death through pyroptosis signaling pathways, trigger the secretion of IL1β, leading to neuroinflammation and worsening AD pathogenesis. Molecular docking verification demonstrated that the predicted natural products scoulerine and genistein displayed strong binding affinities for BACE1 and ESR1, respectively. This strategy can be used to design novel, personalized therapeutic approaches to treatment and a first-in-class clinical lead for the personalised treatment of AD.

## INTRODUCTION

Alzheimer’s disease (AD) is a neurodegenerative condition that first presents with issues around memory loss and gradually progresses to language difficulties, disorientation, behavioral issues, and dementia. The etiology of AD is poorly understood, and treatment options are limited to disease prevention and slowing of symptom progression. Pharmaceutical treatment options have had little benefit. Idiopathic AD accounts for 99% of cases, with only a small minority attributed to the gene mutation associated with early-onset AD [1]. The two characteristic pathological findings in the CNS of patients with AD are extracellular “amyloid plaques” formed by the accumulation of the insoluble protein amyloid-beta (Aβ) and intracellular neurofibrillary “tau tangles” formed by hyperphosphorylation and subsequent aggregation of the cytoskeletal-stabilising tau protein. These two features contribute to chronic neuroinflammation states, leading to neuronal cell death [2].

The formation of extracellular Aβ plaques is considered a neuropathological hallmark of AD and has attracted extensive research [3]. In line with the Aβ hypothesis, one reasonable way to delay AD pathogenesis is by preventing the initial Aβ aggregation of toxic oligomers, fibrils, and plaques. Tau tangles are formed by abnormal phosphorylation and subsequent aggregation of the usually microtubule-related protein, which under normal conditions acts to stabilize cell structure-supporting microtubules, which ensure fast axonal transport and normal cognitive performance [4]. There has been a lengthy and ongoing scientific debate around the causative factors of AD, and the relative importance of both senile Aβ plaques and tau tangles has been largely informed by postmortem investigations of the AD brain. For several decades, the amyloid hypothesis has dominated the field, which has brought forth many high-profile therapeutic attempts that have produced side effects but no real benefits [5]. Thus, a growing body of research has started to re-examine alternative hypotheses, including tau tangles or neuroinflammation, to determine whether they are major pathogenic factors in neurodegenerative and neuroinflammatory diseases [6]. Amyloid deposits can also be a variable outcome and can even present asymptomatically when there is extensive accumulation in the brain [7]. In contrast, tau protein pathology is concentrated most severely in areas related to language and memory [8]. Although the hyperphosphorylation of tau is known to induce aggregation, the mechanisms underlying tau-associated cytotoxicity, cell death, and the phosphorylation site critical for the process remain poorly understood [9].

While there is a long-standing debate regarding the precise cause of AD pathology, a growing body of evidence agrees that altered axonal transport and mitochondrial abnormalities are implicated in the onset and progression of this neurodegenerative disease. Most notably, the AD brain typically shows signs of axonal degeneration, with the signature organelle abnormality (including mitochondria) occurring in large swellings of degenerated neurites [10]. Axonal guidance molecules such as netrins, semaphorins, and ephrins-produced in response to neuroinflammation-were deemed a causative factor in AD progression [11]. Moreover, progressive axonal degeneration was thought to contribute to tau deposit formation and early-stage AD pathogenesis. Furthermore, the deposition and accumulation of tau cause neuroinflammation, which induces irreversible neuronal and cognitive dysfunction in AD through multiple mechanisms [12]. However, exactly how neuroinflammation is linked with neurodegenerative disorders remains to be explained [11]. Recent pioneering research, which conducted positron emission tomography (PET) brain imaging on 130 patients across the aging/AD clinical spectrum, confirmed microglial activation as a critical determinant in the linkage of amyloid plague aggregation to tau spread and, subsequently, cognitive function impairment. The concurrence of Aβ, tau, and microglia activation abnormalities (neuroinflammation) was found to be the strongest predictor of cognitive impairment [13]. Furthermore, the activation of Aβ, tau, and microglia synergistically promotes the occurrence of AD. For the first time in living patients, this research demonstrated that neuroinflammation is the key upstream mechanism crucial to AD development, while secondary infections and new inflammatory events amplify the brain’s immune response and worsen cognition in AD; even in respect of secondary infections which occur outside the brain [14]. In light of this, it is evident that a fuller understanding of the molecular complexity of neuroinflammation may help to identify novel therapeutic targets against the devastating effects of AD.

This study explores the potential relationship between the estrogen receptor-α gene (*ESR1*) and neuroinflammation. Previous epidemiologic studies have shown that AD cases are less common in men than women: a risk significantly enhanced in postmenopausal women. Almost two-thirds of AD patients in the US are female has been attributed to estradiol levels after menopause, which decline to lower levels than those in men [15]. Studies have shown that hormone therapy with estrogen was not associated with an increased risk of developing dementia but a slightly decreased risk [16]. While recent research has further concluded that the more rapid spread (and 75% greater accumulation rate) of tau pathology renders women more prone to AD than men, the precise reason behind this finding was not elucidated [17]. A meta-analysis of regional European differences showed that the Pvull and Xbal variants in the *ESR1* gene might influence the risk for AD by affecting the estrogen receptor expression, and available data have established a clear relationship between estrogens and apolipoprotein E (*APOE*), which represents the foremost genetic risk factor for late-onset AD [18]. We initially attempted to understand the mechanisms underlying breast cancer by identifying the high-throughput dataset of *ESR1* knockdown breast cancer samples [19]. The Kyoto Encyclopedia of Genes and Genome (KEGG) pathway enrichment showed that the most frequently occurring proteins were enriched in axonal guidance and inflammation-related gene hallmarks. However, although a previous study showed that *BARHL1*-*ESR1* network possibly regulates β-amyloid metabolism and memory, the interaction mechanism between *ESR1* and *APOE* is still unclear [20]. AD is characterized by three major questions: Why is age the primary risk factor? Why are women more sensitive to the onset of this form of dementia? And why are neurons in areas of the brain that are essential for memory selectively targeted? By identifying gene expression levels that are common between *ESR1* knockdown breast cancer cells and AD-related neuroinflammation, it may be possible to speculate on the molecular mechanisms underlying the increased risk of AD in postmenopausal women. As such, it is hoped our research may provide increased mechanistic insight at the molecular level associated with the pressing issues of AD and may inform small molecule drug discovery programs to this end.

## RESULTS

### The logFC correction and differentially expressed genes (DEGs)

**Figure 1:**
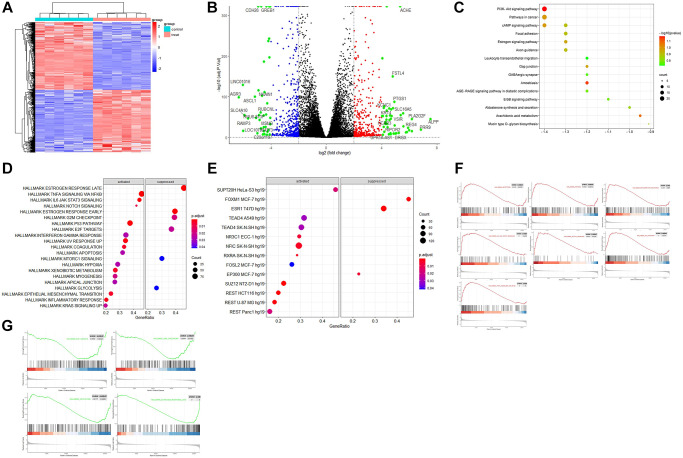
**The bioinformatics analysis of the dataset GSE153250.** (**A**) Heatmap of hierarchical clustering analysis of DEGs between the control (GSM4636683, GSM4636687, GSM4636691, GSM4636695, GSM4636699, and GSM4636703) and treatment (GSM4636684, GSM4636688, GSM4636692, GSM4636696, GSM4636700, and GSM4636704) of siESR1; (**B**) Volcano displays the effect sizes of the control and treatments of GSE153250 with log2 fold change on the x-axis and -log10 adj *p*-values on the y-axis; (**C**) TF enrichment in Hallmark analysis on the activated and suppressed genes; (**D**) KEGG enrichment analysis of DEGs; (**E**) Hallmark enrichment analysis on the activated and suppressed pathways; (**F**) GSEA analysis of activated pathways in the dataset; (**G**) GSEA analysis of suppressed pathways after ESR1 depletion in the dataset.

The principal component analysis (PCA) and signature gene plot confirmed the data qualification. The PCA plots found that the fragments per kilo base per million mapped reads (FPKM) and differentially expressed genes were reliable for the following DEGs analysis. The result of logFC correction is shown in Supplementary Figure 1. The plots of *ESR1*, *AGR3*, *ALPP*, *GREB1*, *SLC4A10*, and *VSIR* demonstrated differences in expression following *ESR1* knockdown (Supplementary Figure 2). As expected, the knockdown resulted in reduced *ESR1* expression. At the same time, the heatmap of the hierarchical clustering of DEGs showed a clear separation between the control and the treatment (Figure 1A). Finally, the volcano plot confirmed the number of upregulated genes as 383 and the number of down-regulated genes as 405, with the cutoff for LogFC at 2. The top 30 genes among DEGs were labeled in the plot (Figure 1B). Finally, the volcano plot confirmed the number of upregulated genes as 383 and the number of down-regulated genes as 405, with the cutoff for LogFC at 2.

### KEGG enrichment, KEGG pathway

The results of KEGG pathway enrichment analysis showed that DEGs were mainly involved in PI3K-Akt signaling, pathway in cancer, cAMP signaling, focal adhesion, estrogen signaling, axon guidance, leukocyte trans-endothelial migration, gap junction, GABAergic synapse, AGE/RAGE signaling pathway in diabetic complications, ErbB signaling pathway, mucin-type O-glycan biosynthesis, and aldosterone synthesis and secretion (Figure 1C). These specific signaling pathways include estrogen, PI3K-Akt, AGE-RAGE, and ErbB, which can regulate inflammatory, neuroprotective, and oxidative effects. The axon guidance (hsa04360) pathway mainly included *BMPR1B*, *EPHA2*, *EPHB1*, *EPHB6*, *PLXNA2*, *PRKCA*, *SHH*, *SLIT1*, *SEMA7A*, *SEMA5A*, *PAK4*, *GDF7*, and *SEMA3D*. In addition, the axonal guidance pathway interacted with the Wnt signaling pathway, which is involved in cell-fate determination, survival, and proliferation. Their interaction and balance might be disrupted in aging and aging-related diseases (Supplementary Figure 3). The GABAergic synapse pathway consisted of *ADCY1*, *HSD3B1*, *LIPE*, *NPR1*, *PRKCA*, *PRKCG*, *CACNA1G*, and *CAMK1G*. AGE/RAGE signaling pathways included *BCL2*, *COL4A5*, *COL4A6*, *EGR1*, *CXCL8*, *PRKCA*, *RNASE1*, *SELE*, and *TNF*. The upregulated genes involved in KEGG maps are highlighted in red, while the down-regulated ones are highlighted in green (Supplementary Figure 3).

### Hallmark enrichment, GSEA analysis, TF enrichment

Hallmark gene sets in the Molecular Signatures Database (MSigDB) collections represent specific and well-defined biological states or processes and offer a coherent expression of the gene sets. The hallmark enrichment analysis found that the activated pathway included TNFα signaling via NF-κB, P53 pathway, IL6 JAK STAT3 signaling, inflammatory response, Notch signaling, hypoxia, and apoptosis. In contrast, the suppressed pathway included mTORC1 signaling, G2M checkpoint, E2F targets, glycolysis, and late/early estrogen response (Figure 1D). The TF enrichment of DEGs from GSE153250 was grouped by the family of the corresponding transcription factors (TF), including both activated (*SUPT20H*, *TEAD4*, *NR3C1*, *NFIC*, *RXRA*, *FOSL2*, *SUZ12*, and *REST*) and suppressed (*FOXM1*, *ESR1*, and *EP300*) (Figure 1E). GSEA revealed significant differences (|NES|>1, false discovery rate, FDR < 0.05; NOM *p* < 0.05) in the enrichment of the MsigDB collections. Therefore, the most significantly enriched signaling pathways based on NES were selected to generate the GSEA plots (Figure 1F and 1G). The GSEA plots sorted the genes according to the degree of differential expression of two samples compared with predefined gene sets (green line indicates activated while red indicates suppressed).

### Validation dataset of AD-related pyroptosis

**Figure 2:**
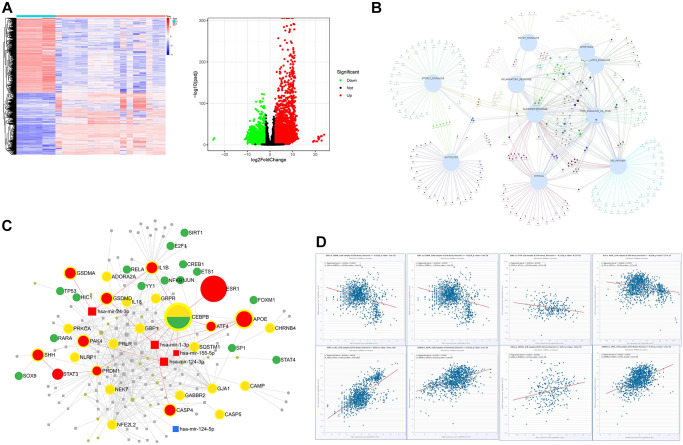
**The bioinformatics analysis of the dataset GSE139549.** (**A**) The heatmap and volcano plots representation of DEGs; (**B**) The interaction of DEGs between GSE139549 and HALLMARK enrichment derived from GSE153250; (**C**) Target gene-TF-miRNA network of the intersected DEGs between HALLMARK enrichment and GSE139549; (**D**) Co-expression of TF or genes in tissues of brain and nerve.

7,249 (2,950 upregulated and 4,299 downregulated) DEGs were screened according to an analysis of the gene expression of samples and data matrix of GSE139549 (*p* < 0.05 and a minimum 2-fold change) (Figure 2A). These DEGs derived from GSE139549 were intersected with genes enriched in the HALLMARK pathway, including Notch signaling, inflammation response, apoptosis, IL6 JAK STAT3 signaling, TNF signaling via NFkB, P53 pathway, hypoxia, glycolysis, and mTORC1 signaling. The Venn diagram confirmed two distinct clusters: 1) suppressed pathway including glycolysis and mTORC1 signaling; and 2) activated pathway that connected closely (Figure 2B). The interaction of HALLMARK genes and genes derived from GSE139549 yielded 2,311 common genes used in the following analysis. The search for pyroptosis-related genes from Genecards was conducted to verify the newly discovered caspase-1-dependent programmed cell death process involved in AD. The intersection of 162 pyroptosis-related genes shared with common genes from the above step was also analyzed. The intersection results found that 25 genes mainly include *APOE*, *CASP5*, *IL18*, *GBP5*, *GSDMA*, *GBP1*, *NLRP1*, *IL1B*, *NEK7*, *CEBPB*, *GJA1*, and *CD274*.

### Protein-protein interaction network (PPI), target genes-TF-miRNA network, co-expression

Based on the STRING database, the PPI analysis of key genes was performed and visualized by Cytoscape. The top 10 genes, including *IL18*, *IL1β*, *P2RX7*, *CASP4*, *CASP5*, *NLRC4*, *NLRP1*, *GSDMD*, *GSDMA*, and *NEK7*, were deemed hub genes in line with the node degree score in Cytoscape. According to topological analysis, such hub genes are highly connected genes in the network and may play an important role in neuroinflammation. PPI analysis showed that *IL1β*, *LI18*, *NLRP1*, *CASP4*, *and GSDMD* were the top 5 targets with a high degree. The data with a confidence score >0.4 were introduced into Cytoscape to construct clustering subnetworks using the MCODE algorithm and resulted in 4 cluster networks. The cluster with a higher score was held to be a more meaningful module in the PPI network. The biggest clustering involved *IL1β*, *APOE*, *GSDMA*, *CEBPB*, *NFE2L2*, *GBP5*, *GBP1*, and *SQSTM1*, followed by the clustering of pyroptosis signature genes including *NLRC4*, *CASP5*, *CASP4*, *GSDMD*, *NLRP1*, and *NEK7*. Twenty-five key genes were imported into the miRNet database to construct the regulatory network of the TF-miRNA-target gene to determine the novel TF-miRNA-target gene feed-forward loop (FFL) model of AD (Figure 2C). The key regulatory network modules included *ESR1-CEBPB-ATF4-APOE*, *ESR1-CEBPB*-mir-155-5p-*APOE*, *ESR1-CEBPB-PRDM1-STAT3-SHH*, *ESR1-CEBPB*-mir-124-3p-*PAK4*, *GSDMD*-mir-1-3p-*APOE*. The key TF included *SIRT1*, *ETS1*, *SP1*, *JUN*, *RELA*, *TP53*, *CEBPB*, *ATF4*, *E2F1*, *YY1*, and *FOXM1*. The key miRNA included has-mir-24-3p, has-mir-124-3p, has-mir-155-5p and has-mir-1-3p (Figure 2C). The co-expression of these key TFs performed in the CHIPbase database showed that there was a negative regulatory relationship between *ESR1* and *CEBPB*, or *ESR1* and *ATF4*, in both 278 samples of GTEX nerve and 1146 samples of GTEX brain (with significant *p* values), while there was a positive regulatory relationship between *ESR1* and *SP1*, or *ESR1* and *FOXM1*, in both 1,146 samples of GTEX brain and 278 samples of GTEX nerve. Positive regulatory relationships were observed between *APOE* and *CEBPB*, *APOE* and *ATF4* (Figure 2D).

### Drug prediction

**Figure 3:**
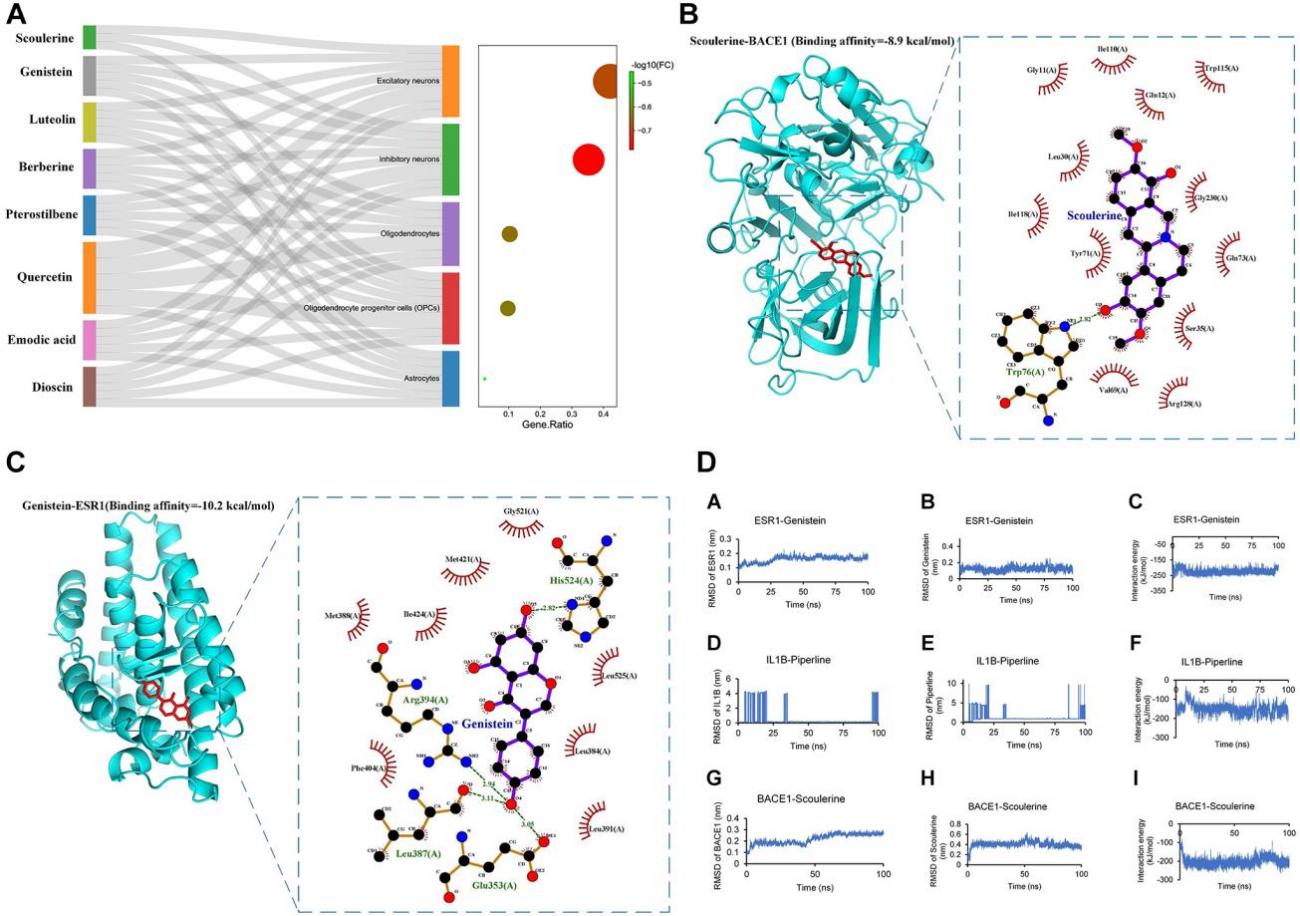
**The identification of candidate drugs and validation of identified drugs.** (**A**) Sankey plot showcasing the association of 8 natural products from CMap with their target subtype cells of single-cell RNA-seq dataset of 81,271 genes. The dot plot showed the gene ratio of each subtype cell targeted by natural products (*p* < 0.05); Natural products (**B**) Scoulerine; (**C**) Genistein) with the highest drug-likeness scores (Table 1) with docking patterns of target proteins (**B**) BACE1, (**C**) ESR1, respectively) according to the lowest binding affinities. The binding affinities (−8.9 kcal/mol and -10.2 kcal/mol, respectively) and binding residues are presented in the Figure. The binding affinity of less than −7 kcal/mol represents a strong binding between the bioactive product and the target protein. (**D**) The molecular dynamics results included RMSD of protein and small molecular and the interaction energy between the protein and small molecular.

**Figure 4:**
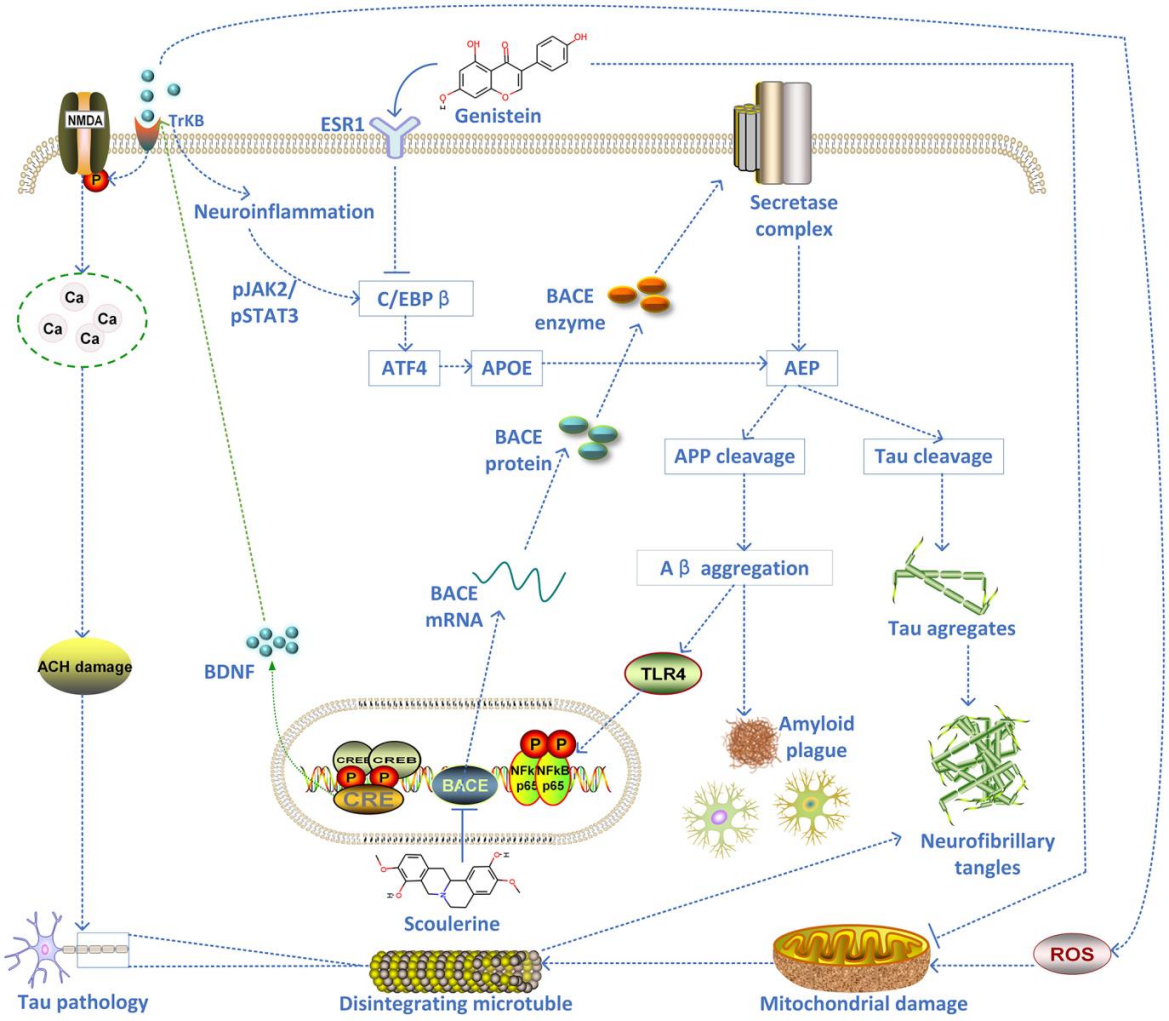
The schematic diagram of the proposed mechanism of two inhibitors of AD pathology (scoulerine and genistein).

The top-scoring 21 natural products with the highest relevance score from Connectivity Map (CMap) results were selected to query their target genes in the SymMap platform or predict their targets in PharmMapper Server or SEA Server (Similarity ensemble approaches) based on their 3D structures downloaded from PubChem database. The targets of these 8 viable natural products were intersected with the biomarker genes of each cell type (5 clusters of scRNA-seq AD dataset). The intersected results were used to build a Sankey diagram to show the combined therapy of these 8 natural products (quercetin, emodic acid, dioscin, pterostilbene, berberine, luteolin, genistein, and scoulerine) on 5 different AD cell types, including excitatory neurons (total 208 genes targeted by these natural products); inhibitory neurons (174 genes); oligodendrocytes (51 genes); oligodendrocyte progenitor cells (OPCs) (48 genes); and astrocytes (12 genes) (Figure 3A). In addition, both scoulerine and genistein have the highest drug-likeness scores, indicating good druggability (Table 1). The molecular docking validation showed that the predicted natural products scoulerine and genistein strongly bind with BACE1 (Amyloid Precursor Protein Lyase 1) and ESR1, respectively (Figure 3B and 3C). In order to verify the stability of the docking structures, we selected ESR1-Genistein, IL1B-Piperline, and BACE1-Scoulerine complex for dynamic simulation analysis. The Root Mean Square Deviation (RMSD) of proteins and small molecules in the complex structures remained relatively stable during the simulation, especially ESR1-Genistein and BACE1-Scoulerine complex (Figure 3D). However, the RMSD of IL1B-Piperline varied greatly. The average interaction energy of ESR1-Genistein, IL1B-Piperline and BACE1-Scoulerine complex was -224.54 kJ/mol, -149.92 kJ/mol and -202.53 kJ/mol. The MOA of scoulerine and genistein was shown in the graphical overview (Figure 4). The literature validation of these two predicted natural products is shown in Table 2.

**Table 1:** The characteristics of natural products from CMap.

**Name**	**MOA**	**Score**	**Druglikeness weight**	**Druglikeness grading**
Scoulerine	GABA receptor antagonis	−1.57	0.886	Good
Emodic acid	Laxative	−1.44	NA	NA
Dioscin	Anticancer	−1.41	NA	NA
Quercetin	Polar auxin transport inhibitor	−1.40	0.506	Moderate
Pterostilbene	Cyclooxygenase inhibitor	−1.38	NA	NA
Berberine	LDL receptor activator	−1.37	0.664	Moderate
Luteolin	Glucosidase inhibitor	−1.36	0.598	Moderate
Quercetagetin	PIM inhibitor	−1.33	0.432	Weak
Genistein	Tyrosine kinase inhibitor	−1.32	0.739	Good

**Table 2:** The literature validation of predicted natural products.

**Natural products**	**Origin**	**Results**	**References**
Scoulerine	Folk medicine *Corydalis cava*	FRET assay, inhibitors at a concentration of 5 μM (24.34 ± 0.36%), IMER assay, 19.02 ± 1.59% inhibition (5 μM)	[52]
Genistein	Soybeans and soy-derived foods	High concentration (25 M) Genistein induces apoptosis pathways by upregulating ESR1 on MCF-7 BC Cells	[54, 73, 74]

## DISCUSSION

ESR dysfunction likely plays a role in AD pathology - especially in women - although the specific mechanisms remain unclear. *In vivo* and *ex vivo* studies demonstrate that neuroinflammatory brain states overlap with ESR signaling pathways and that these two systems interact closely. The majority of neurons and astrocytes in an *IGF1R*-expressing rat brain also express either *ERα* or *ERβ* [21]. Both *ERα* and *ERβ* are broadly distributed in the central nervous system. *ERα* is thought to play a vital neuroprotective role in the context of AD. A reduction in ERα expression has been identified in hippocampal neurons of AD patients (especially females), which has been linked to inflammasome activation triggered by mitochondrial dysfunction and oxidative stress [22]. A growing body of research points to the vital role of ER in maintaining cognitive function across multiple species and paradigms. This is especially true for aging females in the absence of ovarian (or exogenously administered) estrogens. Meta-analysis and clinical studies have also revealed that the variability in *ESR* expression in older women (and men) is associated with variability in the risk of cognitive impairment [15]. Around 80% of postmenopausal females display difficulty with concentration, overreaction, and forgetfulness due to neuronal degeneration caused by estrogen reduction [20]. However, the regulatory mechanisms between the genes involved in estrogen metabolism and the onset of AD are still unclear. Nonetheless, much of the published meta-analysis implies a positive association between the polymorphisms of the gene encoding for ESR1 and the risk of AD in postmenopausal females across such geographically distinct populations as Europe, China, and the USA, suggesting that hormone replacement therapy could alleviate the situation [23]. A growing body of evidence points towards AD development being driven by factors including brain development and distinct gender biochemistry [24]. However, since the regulatory mechanisms underlying sex disparity in AD are still poorly understood, efforts to differentiate AD by sex, rather than pooling data for both sexes, could be an important stepping stone to devising new therapeutic directions for personalised treatment and disease management [25].

The current study initially analysed the relationship between *ESR1* knockdown models and breast cancer pathogenesis at a systematic level. Interestingly, the results showed that *ESR1* knockdown influenced axonal guidance processes, inflammatory activation, and Notch signaling pathways. However, no studies reporting a relationship between *ESR1* and axonal guidance have yet been reported. Furthermore, KEGG pathway enrichment showed that the affected axonal guidance processes are implicated in the proper functioning of the Wnt paracrine and autocrine cell signaling pathways, which are integral for neurodevelopment and the formation and function of intricate neural circuits [26]. Axonal guidance processes allow neurons to determine the optimal direction of growth for their axons to reach desired targets. Axons are vulnerable to decay when excess tau binding to cytoskeletal microtubules impairs axonal transport traffic by impeding motor proteins, which causes synaptic decay even for stable microtubules [27]. The cleaving of *BACE1* is essential to maintain the connectivity of olfactory sensory neurons [28, 29]. Although the causes of AD are still a matter of debate, many mechanisms have been put forward. Axonal guidance molecules have been proposed as participating in different mechanisms underlying the occurrence and development of AD [30]. Neurons depend on efficient axonal transport systems to deliver lipids, proteins, and organelles to the axon and synapse [31]. To function appropriately, axonal transport systems rely on correctly assembling all subcomponents, such as microtubules and motor proteins [12]. Alterations to axonal transport systems may make neurons vulnerable to synapse loss and axonal degeneration [31]. Abnormal axonal morphology and alterations to transport have been found in the early stages of AD. They can be detected up to a year prior to classic AD neuronal pathology, such as amyloid plaques [31]. As such, the axonal transport defects seen in AD have been speculated to be a causative factor in neurodegeneration and have been extensively studied [12]. Tau is a pivotal protein stabilising the microtubule cytoskeleton that functions as an axonal transport track. An imbalance in intracellular signaling leads to excessive tau phosphorylation and, ultimately, tau detachment from microtubules. This, in turn, may trigger microtubule destabilization and axonal transport impairment [31]. As seen in AD, many axonal guidance molecules are upregulated during pro-inflammatory states, including beta-amyloid accumulation, as seen in AD [32]. Axonal guidance molecules can play protective or destructive roles in these states, in line with their receptor-ligand combinations [33]. The over-expression of genes for axonal guidance has decreased neuroinflammation in neurodegenerative disease models [11]. In short, the mechanism underlying these phenomena remains elusive and may implicate various types of programmed cell death, such as apoptosis, pyroptosis, or ferroptosis. Consequently, it remains to be seen whether *ESR1* dysfunction is involved in the newly observed pyroptosis seen in AD. There is evidence that axons have remained intact (one of the characteristics of pyroptosis) even in the final stages of the AD process, despite significant cytoskeletal abnormalities [34]. Since these abnormalities do not change in size, shift in direction, or show signs of re-absorption or degradation, they are likely due to insoluble inclusions of inert and highly aggregated forms of irreversibly hyperphosphorylated tau proteins [35].

The hallmarked pathways related to Notch signaling, TNFα signaling via NFKB, IL6 STAT3 signaling, coagulation, apoptosis, hypoxia, and inflammation, were all activated after *ESR1* knockdown, while those pathways which were found to be suppressed were related to estrogen response late/early, mTORC1 signaling and glycolysis. This result suggests that the *ESR1* knockdown may upregulate a series of pro-inflammatory and pro-coagulation factors, which ultimately promotes adhesion and migration of peripheral leukocytes, activation of the coagulation cascade and disruption to the integrity of the blood-brain barrier [35]. The hyperactivation of Notch signaling may cause neuronal degradation, suggesting a potential role in β-secretase dysfunction in sporadic AD cases. Overexpression of Notch genes in the Notch signaling pathway was reported in AD patients, potentially due to enhanced APP cleavage in AD. Moreover, the Notch pathway can interact with the Wnt pathway, which plays a crucial role in vascular sprouting and regression in angiogenesis that contributes to the pathogenesis of AD [36]. Understanding the molecular cascade related to Notch activation during AD progression may elucidate the complicated signaling network that contributes to the progression of AD. Other studies have found that neural excitotoxicity also upregulates Notch signaling components and thus the severity of AD, which supports the possibility of Notch signaling involvement in post-excitotoxic neuronal demise [37]. Classically, microglial activation induces the expression of toll-like receptor (TLR) and triggers NF-kB-dependent inflammation, and subsequently upregulates inflammatory pathways through cytokines such as IL6, IL12, TNFα, and IL23 [38]. IL23 mediates inflammatory responses by inducing IL17 production and the secretion of pro-inflammatory cytokines. Upregulation of hypoxia-inducible-1α (HIF1α), a protein induced by hypoxia during inflammation, may facilitate AD pathogenesis by upregulating *BACE1* gene expression [38]. Hypoxia and inflammation are intimately linked because hypoxia induces inflammation, while inflamed tissue can become hypoxic [39].

Therefore, neuroinflammation has emerged as a crucial factor in AD pathogenesis [38]. However, the process by which neuroinflammation contributes to the progression of neurodegenerative disorders in aging people, especially in women, remains poorly understood. In particular, the molecular complexity of *ESR1* dysfunction associated with neuroinflammation remains unanswered. Understanding the molecular mechanisms of *ESR1*- associated neuroinflammation and subsequent neurotoxicity may aid in identifying therapeutic targets and provide new windows of opportunity for AD treatment. An independent dataset from high-throughput RNA-seq on AD inflammatory samples was reprocessed as a validation gene set to elucidate critical cellular and molecular mechanisms underlying the *ESR1* dysfunction in AD. The intersection between *ESR1*-related hallmarked genes and AD inflammatory-related genes resulted in essential regulatory genes related to *ESR1*-associated neuroinflammation. *ESR1* and *SP1* are known to transactivate genes that modulate their target genes together [40], such as *Slc2a4*/*GLUT4* expression, which might alter glycemic homeostasis. At the same time, the decrease in *ESR1* activity, failing to counterbalance the *ESR2* action, will also be deleterious to glycemic homeostasis [40].

Caspase-1 is activated upstream in inflammasome NLRP3, which contributes to the maturation of IL1β and IL18 and cleavage and activation of gasdermin D (GSDM*D*). This acts as the pyroptosis executor to release the N-terminal domain, which can cause membrane pores [41] to release intracellular contents, such as IL18, IL1β, and LDH, into the extracellular environment. This release will eventually lead to the occurrence of pyroptosis [42]. Activating transcription factor 4 (*ATF4*) is an Endoplasmic Reticulum (ER) stress biomarker, the accumulation of which can activate *JNK* and retrain Akt phosphorylation. This, in turn, can suppress the phosphorylation of glycogen synthase kinase-3 beta (GSK3β) and activate NOX4/ROS signaling [43]. For the purposes of the present research, *ATF4* was activated in the pyroptosis-induced transcriptional response, and the *ESR1*-knockdown neurons were shown to have an upregulation of *ATF4*. This triggered *APOE* via *CEBPB*. Consequently, the oxide-metabolic driver *ATF4* increased the expression of *APOE* and activated *CASP4* to promote apoptosis and reduce neuronal survival rates. A recent study revealed that CCAAT enhancer-binding protein β (C/EBPβ) plays a key role in the pathogenesis of AD by increasing the expression of asparagine endopeptidase (AEP), and further proposed to activate C/EBPβ/ AEP signaling pathway can mediate AD [44]. In the gene-TF-miRNA co-regulatory network analysis, *ESR1* also mediated the axon guidance molecules (SHH and PAK) through signal transducers and activators of transcription 3 (*STAT3*) via *PRDM1* and mir-124-3p, respectively. *STAT3* is a critical survival signaling factor that enhances the expression of the proapoptotic protein Bax, thereby promoting caspase-dependent apoptosis [45]. The SHH-induced *STAT3* inhibition caused non-apoptotic cell death and, similarly, pyroptosis and necroptosis [46]. The secondary inflammatory challenge of microglia in APP/PS1 mice produces acutely elevated IL1β, which is sufficient to trigger excessive levels of chemokines and IL6 in astrocytes that activate the transcript directly downstream of IL6 signaling (*STAT3*) [14]. Therefore, the notion that the JAK/STAT3 pathway is central in the initiation of astrocyte reactivity is supported by the detection of JAK/STAT3 activation, which is a standard feature of reactive astrocytes [47]. It is also noteworthy that multiple pathways crosstalk to fine-tune the phenotype of reactive astrocytes. For example, STAT3 and NF-kB can physically interact to control target genes synergistically [48]. Nuclear factor erythroid 2-related factor 2 (NFE2L2) is a major TF orchestrating the antioxidant response [49]. The *ESR1* dysfunction also downregulated the E2F target genes, including *FOXM1*, which mediated the *APOE* expression directly [50]. Finally, the target gene-TF-miRNA feed-forward loop demonstrated that the critical regulatory network modules include has-mir-24-3p, has-mir-124-3p, has-mir-155-5p and has-mir-1-3p, which advances our understanding of the molecular complexity of *ESR1* dysfunction induced neuroinflammation as a causative factor of the AD process.

The present study’s application of several bioinformatics methodologies available across different Gene Expression Omnibus (GEO) datasets indicates *ESR1* dysfunction induced neuroinflammation or pyroptosis in the brain and subsequent worsening of the AD conditions. The molecular complexities of AD and potentially diverse research avenues offer a fascinating frontier in biomedical research. In one sense, AD and other neurodegenerative conditions have long been viewed with intense therapeutic nihilism. Currently, there is no cure for AD. Now available therapies can briefly and modestly alleviate symptoms. However, the potential therapeutic molecules and inducer molecules predicted by CMap (L1000) include histamine receptor antagonists, mannosidase inhibitors, progestogen hormones, mTOR inhibitors, retinoid receptor agonists, and cyclooxygenase inhibitors. Interestingly, Ribavirin is an antiviral that can be used as a drug to treat neuroinflammation in AD, targeting *IMPDH1*, *ADK*, *ENPP1*, *IMPDH2*, and *NT5C2*. As such, these drugs should be considered for further verification *in vitro* or *in vivo*. The natural products predicted by CMap were quercetin, emodic acid, dioscin, pterostilbene, berberine, luteolin, genistein, and scoulerine. Among them, quercetin displayed a protective effect against mitochondrial dysfunction and progressive dopaminergic neurodegeneration in cell culture and MitoPark transgenic mouse models of Parkinson’s disease [51] and a protective effect against oxidative stress and brain edema in an experimental rat model of subarachnoid hemorrhage [52]. Both scoulerine and genistein, with the highest drug-likeness scores, demonstrate good druggability. Scoulerine is an effective antimitotic compound and an inhibitor of *BACE1* [53]. The inhibition of *BACE1* can decrease Aβ generation and amyloid deposition; thus, the small molecules with the inhibition effect on *BACE1* are a current focus for AD therapy. After the inhibition of *BACE1* cleavage that is inducible by Sema3A, the CHL1-ntf and CHL1-ctf cannot be released. The CHL1-ctf appears to induce growth cone collapse in thalamic neurons, while soluble CHL1-ntf may interact with neuropilin-1 axon guidance [28]. At the same time, genistein as a soy isoflavone has exhibited numerous health benefits, including suppression of inflammatory responses and anticarcinogenic properties through the modulation of AMPK and COX2 and possibly various MAPKs [54]. Genistein has a dual role in women’s health, which may exhibit a litany of possible biological effects while circulating. Many of its effects stem from its status as isoflavone and, therefore, an estrogen mimic. It primarily acts on estrogen receptors (ERs) via the classical genomic mechanism. Another study reported that genistein downregulates presenilin levels by attenuating ubiquitin 1 expression, reducing Aβ peptide generation and aggregation [55]. As the most common phytoestrogen, genistein showed a strong capacity to bind ESR1 and subsequently can activate or block estrogen receptor ligand-binding domains, thus exhibiting estrogenic or antiestrogenic effects (dual-directional regulation), respectively [40]. The molecular docking verification and molecular dynamics simulation demonstrated that the predicted natural products scoulerine and genistein displayed strong binding affinities with BACE1 and ESR1, respectively, indicating these two natural products (with high druggability) are potential AD drugs. This comprehensive approach has proved a beneficial route to explore various candidate databases such as CMap (L1000) to repurpose existing therapies. More importantly, these findings indicate that anticholinergic drugs might increase the risk of accelerated cognitive decline, especially in older adults at high risk of developing AD. In short, these drug databases can provide essential insights that potentially help deconvolute unknown drug targets, predict inducer molecules, and repurpose therapeutic agents based on the analysis of the dynamic complex network using systems biology approaches.

In conclusion, AD is a biomedical puzzle that continues to attract the interest and curiosity of scientific researchers worldwide. The molecular complexity of AD pathology and the diverse research avenues to approach it present fascinating possibilities for biomedical exploration. Unfortunately, despite enormous efforts, there remains no cure for this terrible illness, and current treatments merely alleviate its devastating symptoms for a short time. This study performed several bioinformatics-based analyses, concluding that ESR1 dysfunction might mediate axonal guidance, induce neuroinflammation or pyroptosis in the brain, and subsequently worsen AD conditions. Cross-validation demonstrated that ESR1 dysfunction could trigger neuroinflammation or pyroptosis as a causative factor in the AD process. The research also leveraged the advantage of CMap as a valuable complementary tool to the phenotype-based new natural compound screening for therapeutic molecules at a systematic level.

## MATERIALS AND METHODS

### RNA-sequencing *ESR1*-knockdown breast cancer dataset and bioinformatics analysis

High throughput analytical techniques and computational analyses enable researchers to conduct large-scale gene expression with high precision. This study was initially devised to identify DEGs for the RNA-seq dataset of the *ESR1*-knockdown breast cancer cells [19]. The raw dataset GSE153250 was selected from the GEO database. The knockdown and control samples were extracted from the siESR and siNT MCF7 cell line in the GSE153250 dataset and analyzed by using the R package Deseq2. Following a normalization and standardization process (PCA examination), the R package was used to correct logFC (log fold change) and collect DEGs between the control (GSM4636683, GSM4636687, GSM4636691, GSM4636695, GSM4636699, and GSM4636703) and treatment (GSM4636684, GSM4636688, GSM4636692, GSM4636696, GSM4636700 and GSM4636704) of siESR1 of GSE153250. LogFC correction is a critical step for the subsequent analysis: especially for GSEA. There are two criteria for screening DEGs by logFC: 1) the threshold of logFC and 2) corrected *p*-value (multiple tests may lead to high false positives). However, genes with small counts but a significant change in expression can skew the logFC. Therefore, the function of lfcShrink was used to correct logFC. The signature gene plot was used to confirm the success of the knockdown experiment. These up-and-down-regulated genes were identified according to logFC and *p* < 0.05. The DEGs matrix was used to perform the following analysis in the study after tidying the data matrix using the R package dplyr [56]. After cleaning the dataset, the signature genes were plotted to test the expression using the R package ggplot2. The heatmap was produced by performing the R package pheatmap. The resulting volcano plot displayed the variation of gene sets in different groups. The ggplot2/ggrepel packages were used to assess the relationship between the *p*-value of a statistical test of each gene.

### Gene ontology (GO) analysis and KEGG pathway enrichment

Based on the general analysis of DEGs, further research, including gene set enrichment, pathway enrichment, and defined group gene set, was carried out to elicit a deeper understanding of genome-based expression. Identifying hub genes and key pathways from common DEGs is essential since finding drug targets frequently depends on such hub genes. Since gene ontology (GO) analysis of DEGs can effectively identify the characteristics of differential gene subsets, GO analysis was performed using EnrichGO in the R package clusterProfiler [57]. In addition, KEGG pathway enrichment was carried out using the function EnrichKEGG in R package clusterProfiler.

### Hallmark enrichment analysis, enrichment analysis of GSEA, TF enrichment

Gene Set Enrichment Analysis (GSEA) was performed using the gseGO, gseKEGG, and gsePathway functions of the R package clusterProfiler. The GSEA method can be applied for analysis and calculations to ascertain whether a priori-defined group of genes has a consistent and statistically significant difference between two biological statuses [58]. As such, GSEA can detect the expression change of gene sets rather than individual genes. Moreover, subtle enrichment detection renders the result more reliable and flexible than the traditional pathways enrichment analyses of GO and KEGG [58]. Gene sets with a normal *p*-value < 0.05 and FDR (false discovery rate) <0.05 were significantly enriched. GSEA enrichment of differential expression signatures of the identified group was carried out for the gene signatures of samples. Positive scores indicate strong consistency [59]. The HALLMARK collection of gene sets downloaded from the Molecular Signature Database (MSigDB) was used for this analysis [60]. Therefore, GSEA revealed significant differences (|NES|>1, false discovery rate, FDR < 0.05; NOM *p* < 0.05) in the enrichment of the MsigDB collections.

### Validation using an independent external dataset of AD neuroinflammation

An independent test set validation was obtained from RNA-seq (smart-seq) on AD inflammation samples and Genecards [61]. The AD-related inflammation data acquired from GEO was generated by reprocessing the dataset of GSE139549 via the same approach used in GSE153250. Combining the RNA-seq and Genecards could effectively expand the sample size and improve the statistical power of detecting DEGs for AD-related pyroptosis. The intersection of the *ESR1*-knockdown HALLMARK dataset (GSE153250) and AD’s inflammation dataset (GSE139549 plus Genecards) yielded the intersecting genes related to AD’s pyroptosis. These essential intersecting genes were used in the following steps to perform protein-protein network analysis and Gene-TF-miRNA regulatory network construction.

### PPI, GENE-TF-miRNA network, co-expression of TF or genes in tissues of brain and nerve

A protein-protein interaction (PPI) network was built based on DEGs using the STRING 11.5 database and visualized by the Cytoscape software [62]. Briefly, the intersecting genes were imported into the STRING database, and the PPI network was constructed with default conditions. The cutoff value was defined as an interaction score of 0.4 (median confidence). The interaction result was imported into Cytoscape to identify gene clusters in the MCODE plug-in unit. The clusters with the greatest number of imported genes were extracted from the results of Cytoscape for further analysis. A target genes-TF-miRNA regulatory network was built in the miRWalk 2.0 database [63]. The selected genes targeting miRNAs were predicted using miRWalk, miRbase [64], and the TargetScan database [65]. The miRNAs validated in these databases were chosen as the predicted results. The selected parameters were set to *p*-value < 0.05, the length of the minimum seed sequence: 7mer and the binding region of the target gene: 3′UTR. According to the website tutorials, the selected miRNA and genes were imported into the miRNet 2.0 database to predict the interactions between miRNA, TF, and genes [66]. The co-regulatory network of Target genes-TF-miRNA was constructed to elucidate the complex regulatory mechanism of pyroptosis-related neuroinflammation in AD. The co-expression of key TFs derived from the Target genes-TF-miRNA regulatory network was analyzed in CHIPbase [67].

### Identification of candidate drugs and validation of identified drugs

The hub genes were used to predict the potential therapeutic and inducer molecules in CMap L1000. This online platform for finding disease-gene-drug relationships is the most comprehensive transcriptome database for potential drug exploration [68]. In this case, a negative connectivity score represents a therapeutic drug. As such, FDR < 0.05 was used to screen molecular compounds, which could potentially reverse the altered expression of DEGs in MCF7 cell lines [69]. The top-scoring 20 natural products with the highest relevance score from CMap results were selected to query their targets in the SymMap platform or predict their targets in PharmMapper Server or SEA Server (Similarity ensemble approaches) based on their 3D structures downloaded from PubChem. The targets of these natural products were intersected with the biomarkers of each cell type, including excitatory neurons, inhibitory neurons, oligodendrocytes, oligodendrocyte progenitor cells (OPCs), and astrocytes. These 5 clusters of the scRNA-seq dataset were downloaded from the SC2disease database about AD early vs. lately onset (81,271 upregulated- and downregulated genes). The drug-likeness was predicted by an Encyclopedia of Traditional Chinese Medicine (ETCM) which has thorough information about bioactive components [70]. Furthermore, two drug molecules with a significant *p*-value were selected to verify by molecular docking software (Autodock vina 1.2.0) [71]. The docking patterns were visualized by LIGPLOT v.4.5.3 [72].

### Molecular dynamics simulation

The molecular dynamics simulations of these complexes were performed using Gromacs 2020.1, in which the charm36-jul2020 force field was chosen. The complex was solved in TIP3P water and immersed in a dodecahedron box extending to at least 1 nm of the solvent on all sides. The system was neutralized by Na^+^ and Cl^-^, then added 0.15 M NaCl. The system was minimized by using the steepest descent algorithm for 5000 steps and made a maximum force of less than 1000 kJ/mol/nm. Then, it was equilibrated in a constrained NVT (number of particles, volume, temperature) and NPT (number of particles, pressure, temperature) running for 100 ps. The system was well-equilibrated through NVT and NPT equilibration at 300 K and 1 bar. Finally, MD simulations of the complex were carried out for 100 ns. The Verlet cut-off scheme and a Leap-frog integrator with a step size of 2 fs were applied. The final analysis of molecular dynamics included RMSD of protein and small molecular and the interaction energy between the protein and small molecular, which were calculated by GROMACS 2020.1.

## Supplementary Materials

Supplementary Figures
