# Supplementary material for: ESR1 dysfunction triggers neuroinflammation as a critical upstream causative factor of the Alzheimer’s disease process
Source: Aging (Albany NY). 2022 Nov 1;14(21):8595–614. doi: 10.18632/aging.204359 (PMC9699767; doi:10.18632/aging.204359)
Supplement: Supplementary Figures [file aging-14-204359-s001.pdf]

SUPPLEMENTARY FIGURES

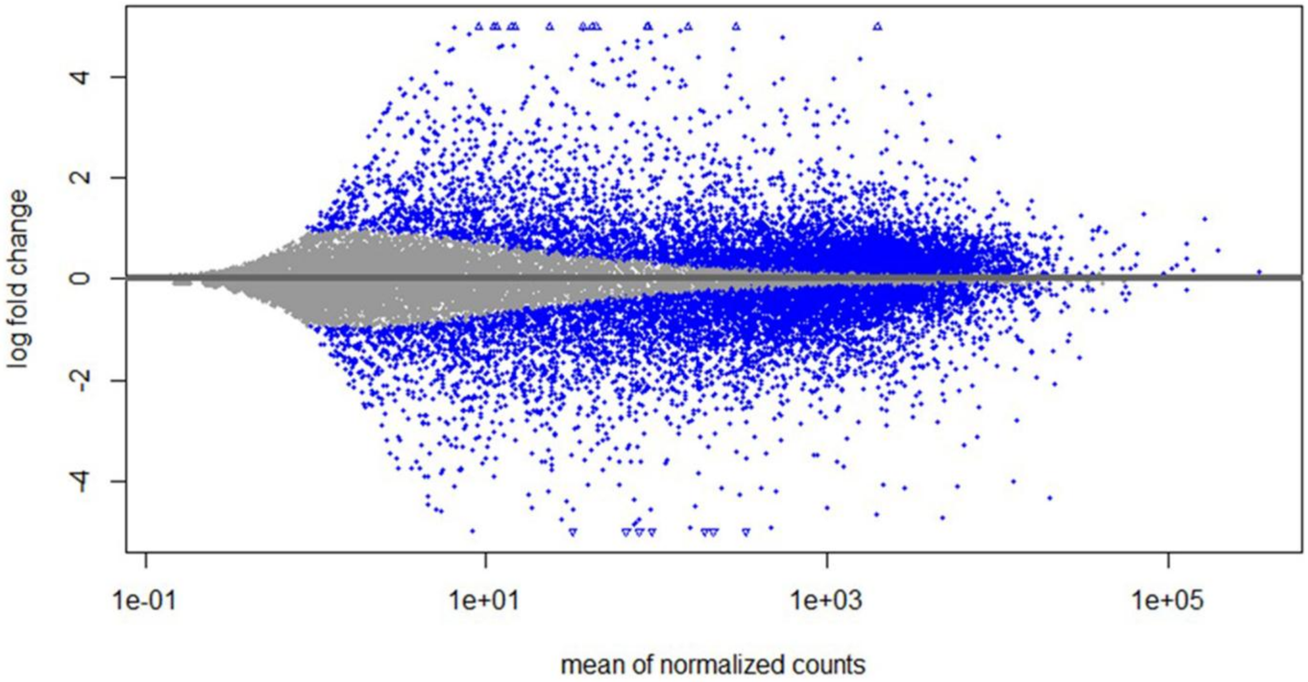

Supplementary Figure 1. LogFC correction of DEGs from the dataset siESR1 of GSE153250.

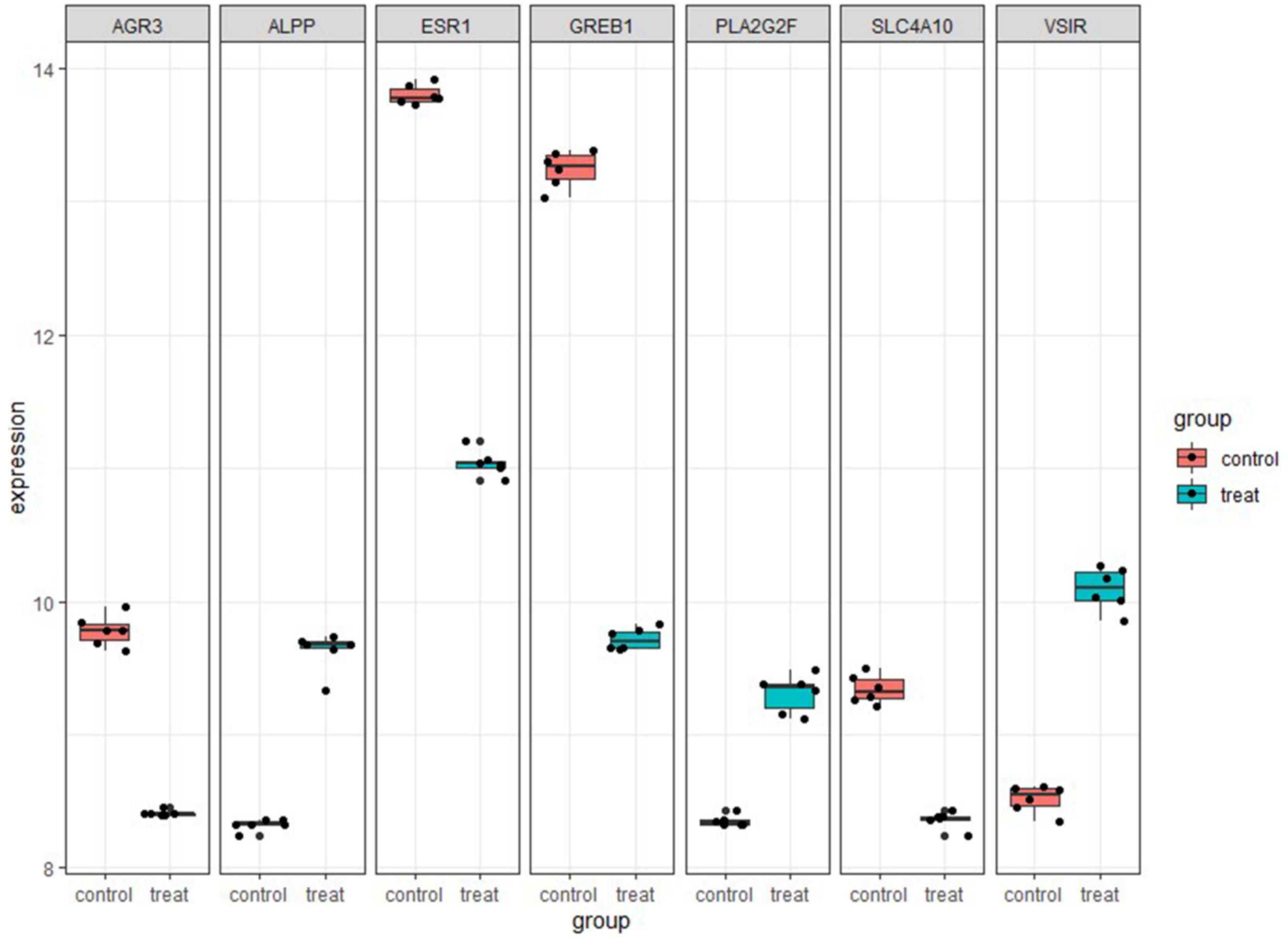

Supplementary Figure 2. The expression of selected DEGs from the dataset siESR1 of GSE153250.

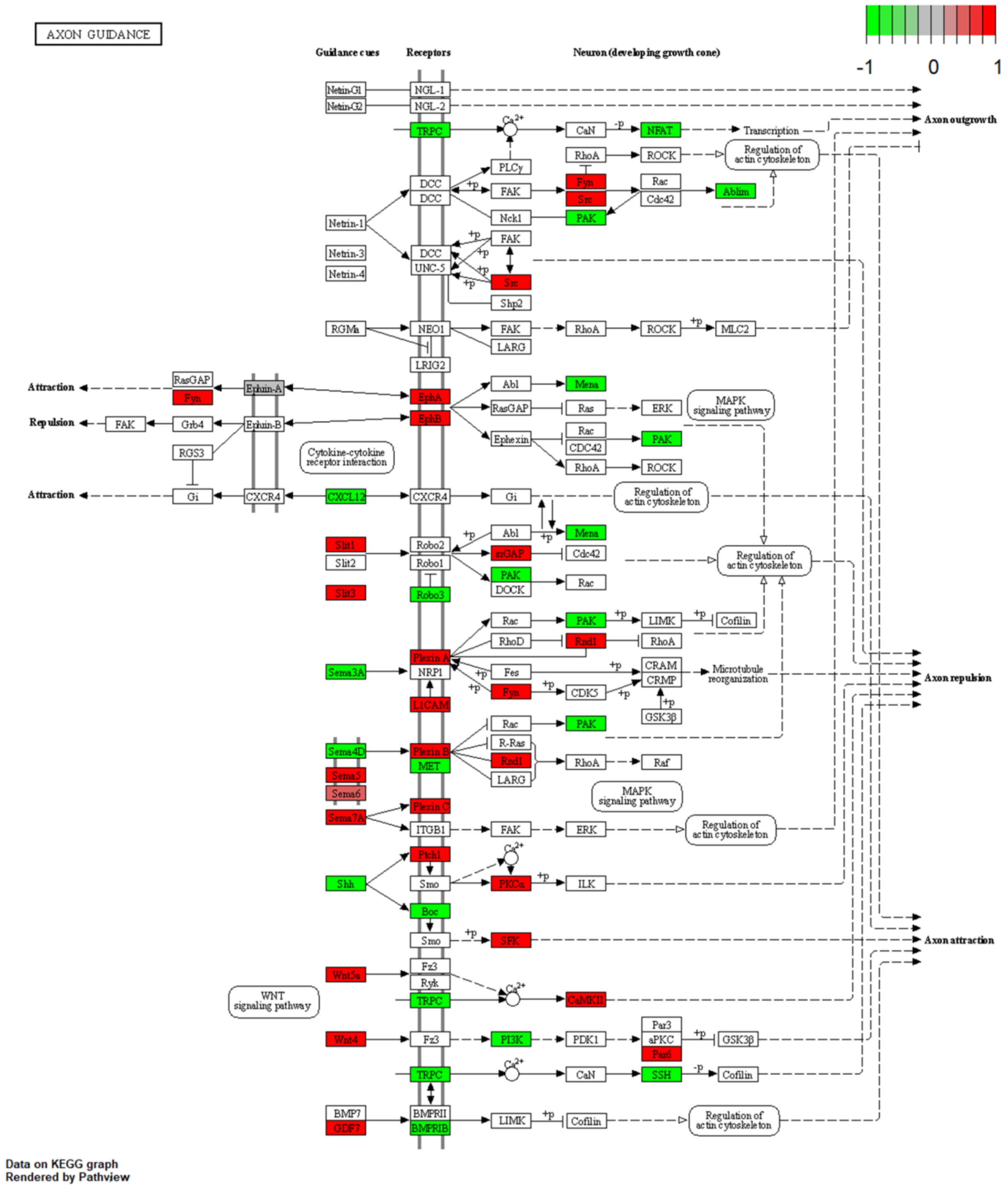

Supplementary Figure 3. KEGG pathways of hsa04360 based on the analysis on GSE153250.
